# The experience of primary care teams during the early phase of COVID-19: A qualitative study of primary care practice leaders in Ontario, Canada

**DOI:** 10.1186/s12875-022-01907-4

**Published:** 2022-11-22

**Authors:** Catherine Donnelly, Christine Mills, Sandeep Gill, Kavita Mehta, Rachelle Ashcroft

**Affiliations:** 1 Queen’s University, 31 George Street, Kingston, Ontario K7L 3N6 Canada; 2 Association of Family Health Teams of Ontario, 400 University Avenue, Suite 2100, Toronto, Ontario M5G 1S5 Canada; 3 University of Toronto, 246 Bloor Street, Toronto, Ontario M5S 1V4 Canada

**Keywords:** Primary care teams, interprofessional primary care, COVID-19

## Abstract

**Background:**

The COVID-19 pandemic has caused a rapid shift to virtual care in primary care practices around the globe. There has been little focus on the experiences of interprofessional teams through the lens of primary care practice leaders. The objective of this study was to examine the experience of primary care teams during the first wave of the COVID-19 pandemic from the perspective of primary care leadership.

**Methods:**

Qualitative study using qualitative description methods. Executive Directors of interprofessional primary care teams belonging to the Association of Family Health Teams of Ontario (AFHTO) were invited to participate. Executive Directors were interviewed and the interview transcripts were analyzed using thematic analysis.

**Results:**

Seventy-one Executive Directors from across all regions of Ontario were interviewed for the study, representing 37% of the AFHTO member clinics. Four themes were identified in the data: i) Complexities of Virtual Care, ii) Continuation of In-person Care, iii) Supporting Patients at Risk, and iv) Stepping up and into New Roles.

**Conclusions:**

Primary care teams rapidly mobilized to deliver the majority of their care virtually, while continuing to provide in-person and home care as required. Major challenges to virtual care included technological infrastructure and unfamiliarity with virtual platforms. Advantages to virtual care included convenience and time savings. Virtual care will likely continue to be an important mode of primary care delivery moving forward.

## Background

COVID-19 resulted in an immediate and profound disruption to primary care [1, 2]. Emerging research has shown that patients continued to receive primary care services despite a rapid shift to virtual care [2–4]. Not only did the mode of care delivery change, but the types of conditions and issues seen by primary care teams changed as well, with a greater emphasis on mental health issues and less focus on chronic physical health conditions [5]. Primary care teams are particularly well suited to support complex issues emerging during the pandemic where the perspectives of multiple disciplines are needed [6, 7].

To date research on primary care during COVID-19 has largely focused on the experiences of physicians and less focus on the broader impact on the teams [8, 9]. One paper has offered the perspective of the interprofessional primary care providers [7]. Missing from this growing literature is that of the primary care practice leaders, such as executive directors. The role of executive directors in primary care clinics is broad, and they are responsible for all the day-to-day management of a primary care team. Depending on the size of the team there may be additional support for financial or program management or in smaller clinics the executive director may be responsible for all operations [10]. A leadership lens offers a broader picture of the issues that primary care teams are facing and takes into consideration the multiple influences on primary care practice, including health care policies, funding, patients, and providers. A recent abstract was published on the findings of a qualitative study examining the perspective of 15 primary care leaders in the United States on the challenges and opportunities of leading during the COVID-19 pandemic [11]. The key themes identified the importance of primary care leaders in supporting their teams manage continually changing environments and the importance of being present and supporting providers [11]. No details of the study were provided, and it is unclear if any of the leaders included in the study worked in an interprofessional primary care model. The research presented in the abstract specifically examined leadership roles and no research has been conducted that explores how interprofessional primary care teams experienced the first wave of the COVID-19 pandemic from the perspective of the leaders. This unique perspective can offer a broader look at the issues teams faced, the decisions that were made and the policies and the role of the teams within the larger system.

The purpose of this study was to examine the experience of interprofessional primary care teams during the first wave of the COVID-19 pandemic from the perspective of primary care leadership.

## Methods

### Study Design

We used a qualitative description study design. Qualitative description is an approach designed to “offer a comprehensive summary of an event in the everyday terms of those events” [12, 13]. This method offers direct answers to questions that are relevant to both practitioners and policy makers by providing a rich description about a phenomenon of interest. Qualitative description therefore was felt to be ideally suited to understand practice changes in interprofessional primary care teams due to the COVID-19 pandemic. The research sought to answer three questions: i) How are interprofessional primary care teams transitioning to virtual care in response to COVID-19?, ii) How are interprofessional primary care teams providing in-person care and care for at-risk patients during the COVID-19 pandemic?, and iii) How are interprofessional primary care teams envisioning the future of primary care delivery after COVID-19?

The interdisciplinary research team partnered with the Association of Family Health Teams of Ontario (AFHTO) to conduct the study, working collaboratively to develop the research questions, facilitate data collection and knowledge translation. AFHTO works to support and advocate for interprofessional primary care teams in the province of Ontario, Canada, with membership of 191 interprofessional primary care teams. We obtained ethics approval from the University of Toronto Research Ethics Board (REB Protocol #39432).

#### Sample

All the Executive Directors (EDs) at 191 primary care teams belonging to AFHTO in Ontario, Canada were invited to participate in the study. AFHTO membership includes Family Health Teams, Nurse Practitioner-led Clinics, Indigenous Primary Care Teams and other team-based care models and are located across the province of Ontario. As per qualitative description we sought maximal variation and therefore recruited all ED’s in the province in order to obtain their perspectives. Interprofessional primary care teams are modeled off the Patient Medical Home [14] and provide comprehensive interprofessional primary care services to approximately 25% of the population of Ontario [15]. These interprofessional primary care teams include a variety of providers, including physicians, nurse practitioners, nurses, registered dietitians, social workers, occupational therapists, psychologists, pharmacists, chiropodists, and other interprofessional health care providers. This study used purposive sampling whereby an email with information about the study and inviting participation was sent to each of the 191 EDs. EDs interested in participating in this study sent an email expressing interest to the study coordinator (SG).

#### Data Collection

We conducted semi-structured interviews to explore how interprofessional primary care teams in Ontario continued to provide care during the COVID-19 pandemic. Informed by emerging literature on the COVID-19 pandemic as well as results from a surveys conducted with interprofesional primary care providers and EDs [7, 16], we developed a semi-structured interview guide. The interview guide was pilot-tested by the research team. One-on-one interviews were conducted between July and September 2020. Interviews scheduled for up to 1 hour, however not all interviews required the full hour. All interviews were video recorded using Microsoft Teams and were immediately transcribed into text verbatim. Only interviewers and the ED were on the video call and each ED was interviewed once. Interviewers were all female and completing a graduate-level health professional degree at the time of the interviews. All interviewers received training on qualitative interviewing and had completed research methods coursework. There was no prior relationship between any of the interviewers and the EDs.

Immediately after each interview, the interviewers created field notes. In particular, the any items that stood out of the following themes was noted: initiatives the team has taken on, community partnerships the team has developed, challenges the team faced, and any additional comments. These notes helped inform the data analysis.

#### Analysis

Data analysis occurred following completion of all interviews. Using thematic analysis [17, 18], two authors (CM/CD) analyzed the data following the steps provided by Braun and Clarke [18]. Thematic analysis is a qualitative descriptive approach that is used to identify, analyze, and report patterns, called themes, within data [17, 18]. We used an inductive approach, whereby the themes identified were linked to the data and informed by the interview guide. Two of the team members (CD, CM) independently conducted a line-by-line review of one of the transcribed interviews. The team members (CD, CM, RA, SG) came together to discuss the early ideas of the data and develop initial codes. From the first discussion an initial code book was developed.. Next, a portion of the transcripts were reviewed by one of the team (CM) and then brought to the larger group (CD, CM, RA) for review where they were discussed until consensus was reached and a master codebook was developed. Each team member was then assigned a group of transcripts to analyze. Data saturation was reached before all transcripts had been analyzed, with new transcripts repeating the same themes previously identified. One of the authors (CM) examined the coded transcripts for strong illustrations of the themes which were then discussed, compared and contrasted and further refined through discussion with three of the other authors (CD, RA, SG). The primary coders, with assistance from the research team, identified exemplar quotes that help illustrate the key themes. We used NVivo 12 (QSR International, 2020) to help organize the data analysis process.

The two authors (CM/CD), who completed the thematic analysis are women who are interprofessional health care providers and who have previously worked in interdisciplinary primary care teams. They are both interested in how interprofessional teams operate, how these teams have dealt with the COVID-19 pandemic, and how the work of interprofessional primary care teams can be improved. All the authors have experience working with interprofessional primary care teams and conducting research on interprofessional primary care teams.

## Results

Seventy-one EDs, from all regions across the province Ontario participated in the study, representing 37% of the AFHTO member clinics. The EDs that did not participate did not actively refuse to participate but did not accept the invitation to participate. As these EDs did not contact the researchers, it is unknown why they did not participate. Four themes were identified in the data: i) Complexities of Virtual Care, ii) Continuation of In-person Care, iii) Supporting Patients at Risk, and iv) Stepping up and into New Roles.

### Complexities of Virtual Care

The province of Ontario instituted an emergency lockdown on March 17th, 2020 [19] to control the spread of the novel coronavirus SARS-CoV-2. Practically overnight, interprofessional primary care teams transitioned from delivering care in-person to virtual, for all but the most urgent and critical needs. Synchronous virtual care included telephone, secure messaging, texting and video appointments as described by one participant: “when COVID-19 hit we were able to transition immediately, within a week. Everybody was, all the providers were using virtual care, and they were working from home. So remote access, telephone encounters, which they’d never done and the video encounters as well.” (P098).

This change to virtual care came with both challenges and benefits, both to providers and to patients. The impact of virtual care was complex and experienced differently by different people.

In general, teams were able to adapt and find solutions to many of the difficulties they encountered.

For many primary care teams, technology presented a significant challenge. For teams in rural, remote, or Northern areas, connectivity was a major issue. “Where we struggle is the connectivity because we’re rural …. In the office we are pretty much okay, but again, it’s not always reliable and … a lot of our patients are in rural areas.” (P065).

One ED reported, “the EMR vendor I’ve called them a couple of times to ask if we can move to the cloud supported service for the EMR and when I tell him our upload and download rates- the last time I told them a year ago, he laughed at me. They were so bad, he was like ‘yeah no, don’t call back we won’t be able to support you moving to the cloud.’ So, it’s really- it’s different and we’re not even remote, we’re rural … we’re Southern Northern Ontario.”(P027) This was a common experience for rural, remote, and Northern teams.

These teams tended to use the telephone for appointments, as opposed to other modes of virtual care, “We have the options for both virtual and telephone, but again we’re rural so a lot of our patients don’t have a good Internet connection, so we’ve been relying on telephone visits.” (P065).

In addition, certain patient populations had difficulties accessing technology. For example, some patients lacked technology, “I think of our patients who maybe don’t have a phone card, they don’t have a laptop or a tablet or any-anything to connect … it shouldn’t be a penalty to them that they can’t access virtual care because their community or their own personal circumstances are prohibiting that.”(P052) Other patients were less comfortable with using technology needed for virtual care appointments, “because of the age of our population, technology is not always their friends, some are very good at it, but most are not.”(P024).

For providers, there was a learning curve in moving to virtual care. One ED stated “The biggest challenge was just for staff. It was a big learning curve … we’re used to seeing patients face to face … We all got put out of our comfort zone … It took … everybody a few weeks I would say to get into the groove of how best to perform a telephone assessment.”(P064).

Some providers found that providing virtual care was more time-consuming: “what I’m hearing from the providers is it’s taking a lot of time.”(P055) Sometimes multiple visits were needed, “I think there been times where our providers have provided two, three phone calls and ends up seeing that individual.” (P076) Another ED reported, “I do hear from clinicians who have longer appointments. So, like say, our mental health clinicians and our dietitians, that there is a higher fatigue level that comes with virtual appointments. Maybe that feeling that you have to work a little harder to kind of track the patient because they’re not in front of you. You can’t always read, especially if they’re telephone appointments you cannot - you know you can’t then read body language or you know, get some of the other cues that you might get.” (P077) Thus, some providers found virtual visits more challenging than in-person visits.

In contrast, other teams found that virtual care took less time and provided for more timely care. Some providers were able to stick closely to their schedules. “The wait times were overall more productive in many respects and have been able to stick to our schedules more closely.” P010) Another ED felt virtual care was more efficient: “it’s a more efficient way to practice medicine for both physicians and patients.”(P062).

Because virtual care was so new, and no existing structures were in place, security and privacy concerns were raised. “There being a lot of privacy concerns … It was quite the drastic change for us. Basically, overnight to say, ‘Woah, what are we going to do?’ … What capacity does our EMR have to be emailing patients securely, because there is a secure portal that we haven’t used, we’ve just stuck with what has worked for us.”(P073).

Ensuring providers had access to technology to work from home was also raised, “first of all, we didn’t have enough resources, we don’t have limitless laptops to set people up at home. P068.” This was echoed by others, “there is no capital budget for equipment.”(P078) Another ED stated, “we had to purchase a number of laptops in order for people to have that flexibility to work from the office part of the day and then work from home the other part of the day.”(P030).

The cost of virtual care platforms was another issue for primary care teams. “Our EMR Vendor, they offered … four months of free virtual visits … Everyone on our team jumped on that and for four months we’ve been using that and suddenly after four months you’ve got to start paying. For physicians … they’re very conscious around overhead costs, most of our physicians said no, I’m not continuing on with this and paying for this.”(P017) Another ED said, “there’s a lot of discussion about Zoom initially and having Zoom availability. The cost of Zoom to our physician team was extravagant in the amount of patients that we would service so that wasn’t feasible.”(P018) However, the importance of having video and being able to physically see a patient was also emphasized. “People still need physical examinations … and over the phone, they can’t see the surroundings, they can’t see if they look tired or they are unwell, if they are unkept … It’s very crucial to have that. But it also can be a costly solution to some teams.”(P040).

Some EDs mentioned that virtual care made it difficult for providers to work together as a team. “It’s really challenging to provide team-based delivery care without having … some opportunities for shared interaction together on-site.”(P076) Another ED stated, “one of the downsides is that the benefit of Family Health Teams is interdisciplinary care. People still connect with each other. We’re all on the same EMR and they send tasks back and forth and they can still phone people. But I’m guessing it’s not happening quite as regularly as it would when people are here in-person.” (P042).

EDs recognized that working from home also presented challenges for providers. “You think of dietitians or social workers, they’ve got children at home. They’ve got a dog barking. Having that patient see into their home. Having the privacy or vice versa.”(P061) Another said, “in rural Ontario … we have some patients where we’re not able - even some providers - who can’t work from home because they don’t have that ability to have a stable, reliable Internet access.”(P032).

Group program participation rates remained high, “normally when you have an in-person group setting as we did previously, the participation would start off great and then would just peter off. What we found is that participation levels were staying in the double digits.”(P031) These levels of participation were linked to the convenience and access of the virtual format. One ED stated, “One of the programs we did launch - and it was an extension of a mindfulness yoga-based therapy program that we were delivering at community centers - and we moved it online.”(P010) Also, “we started doing an anxiety relief group and it’s a 6 week - 6 session program once a week and I had my doubts about whether it was going to work with that particular patient population. I think it’s in week three now and I’m amazed.”(P042).

Focusing on current realities was important for several teams: “our virtual programming, we tried to develop the programs specific to what’s happening now, people being at home and working from home. That’s what we did, our ergonomics program. One of the dietitians is doing a program about intuitive eating and eating when you’re bored because people are at home more. We tried to focus it on what’s happening now as opposed to, previously our programming was what are the things that are important for our Family Health Team in our strategic plan.”(P038).

There were many programs that went virtual: “our pharmacist and social worker just completed a sleep therapy program where they created a secure environment for the patient to submit their sleep records... you’ve got your allied health at home … and they’re busily transitioning how they deliver that program in person to allow online format. The feedback they got from the patients was excellent on every account. Both in terms of our anxiety, depression, sleep therapy, and all of our maternal health programs. The allied health have just stepped up, reconfigured their teaching and learning groups to online and made it work.”(P009).

However, EDs acknowledged that virtual care was not always appropriate. “So, what we’re finding is that virtual works for some people. It does not work for people who have had trauma.” (P099) Concerns about clients with mental health issues was a common theme. One ED articulated concerns that had been express by her team “with the adults mental health, sometimes home is not a safe place, so doing a counsel from home when maybe the person that’s contributing to your mental health issues is in the same room as you is a bit tricky.”(P013) However, others reported their team found that virtual care made mental health visits easier. One ED said, “even with social work, which actually surprised me because if I was meeting at therapist, I thought I might like to see them in person. In some ways the phone provides another level of privacy, and it’s easy.”(P042) Another stated, “we have a social worker … [who] had very positive experiences with providing mental health visits virtually. …. She’s feeling like they [patients] feel that they have a higher sense of anonymity, and she’s having some patients be more open with her through this means.”(P064).

While virtual care presented barriers for some patients, it made care more accessible for others during the COVID-19 pandemic. One ED reported, “I think using virtual care has become a more patient-centred approach … some of the things that we could never overcome in our particular areas such as transportation, now no longer is a barrier to care because patients can be seen.”(P017) Another stated, “All of these barriers are just removed, and people are just like yeah I can jump on my laptop or my phone and just … be there and have that access and speak with somebody … The ease of access is huge.”(P058).

Convenience was also another major advantage. “I think patients are really liking the convenience that telephone and virtual calls have to offer.”(P012) Younger patients in particular found virtual care preferable to in-person, “the younger population prefers virtual. They love the idea that they can still connect and do so in the comfort of their home.”(P036) Patients appreciated not having to commute to the clinic. “Moving to a virtual care environment … we’ve been able to mitigate that commute time for the patient.”(P010) Another ED commented, “some patients have really enjoyed not having to come in. Some of our patients live over 45-minute drive from our practice.” (P027).

EDs reported fewer no-shows for appointments. “Our no-show rates have actually declined. … and patients really appreciate the ability and the convenience that virtual care accords.”(P010) The decrease in no shows was noted “particularly with the allied health, their no-shows reduced … and had quicker follow-up.”(P076).

With this new-found access and convenience patients are already looking to the future. “They’ve expressed concerns that they won’t be able to continue on with the virtual visits, because they’ve had a lot of success with that, a lot of decrease in the no-shows.”(P065) So, while the initial shift was rapid and not always smooth, the benefits are being experienced by many and virtual care is very likely here to stay.

### Continuation of In-Person Care

While virtual care was the primary mode of delivery, some in-person care continued beyond urgent needs. One ED commented, “The nursing staff … agreed to still be on site about two days a week … so that they were still delivering virtual care … [and] if patient needed to be seen for whatever reason they deemed essential.”(P031) This onsite care was for specific issues deemed important for face-to-face. For example, for peri-natal care, “our team has two physicians they are predominantly OB practices. We continue to see all prenatal care and well-baby checks throughout the pandemic. We still had a number of patients coming into the office for immunizations and things like that.”(P065).

In-person visits also continued for those considered high risk of hospitalization “we identified who our high-risk patients were and said these are patients that we can’t manage virtually. These are patients that we physically need to lay hands and eyes on to be able to manage effectively. To reduce the risk of them ending up in acute care facilities. “(P036) In-person appointments also continued for others, “we also saw, a lot of our vulnerable patients, that couldn’t be seen on a video call because they just didn’t have sort of the insight or ability. We continued to see them in the clinic … These appointments were really important, and in a number of cases, whether it was a home visit or the person coming to us, their health had deteriorated to such an extent that we sent them to the emergency department. Very, very important to monitor this population, which is … 20% of our practice.”(P044).

Creative solutions were implemented to allow certain types of care to continue. For example, for nicotine replacement therapy (NRT), “we did a drive through NRT drop off. Our NP arranged a time for them to come to pick up their NRT. It was dropped through an open window into their car or through the window into their bag.” (P048).

Home visits also continued for some patients, such as those who were palliative: “there are still home visits, but those are mostly saved for palliative patients. If … a physician had a palliative patient, there were home visits.”(P065) Other teams increased the number of home visits to specific patient groups, “we have increased home visits for older population and people with chronic disease.” (P025) Different teams, on the other hand, decreased the number of home visits “we scaled down on the number of home visits that we were doing, but there are some patients who we had to do home visits for because they are homebound, they can’t get out of their home. Those patients we tried and maintain that contact with them, … and sort of monitor this situation as what the risk is like in our local settings.” (P002).

For home visits, it was important to have the right policies and procedures in place to ensure the safety of patients and provider as well as securing adequate personal protective equipment (PPE) “we have a physician and NP, part of a nurse that does that do home visiting, and it’s a really important team because all these seniors are homebound. We had to take a really close look at the criteria for who would get a home visit and we had to establish clear protocol around PPE. What we ended up being able to do because we had the PPE, is anybody, particularly the palliative care for the people that were at end of life, we needed to be able to continue seeing these families of those people.” (P037).

### Supporting Patients at Risk

Teams continued to support populations at risk in many ways, such as reaching out proactively to patients. As one ED stated, “in terms of our vulnerable population, we’ve set up a system whereby, nurses regularly call these patients to just do a health check to make sure they’re alright.”(P010) For isolated patients, these wellness checks were particularly important. “because we have some patients who are fairly remote in terms of access to transportation, they found these wellness checks were very, very comforting to them. Sometimes that was really their only access to any social interaction.”(P064).

A number of different patient populations were identified as priorities. “We definitely focused on patients that had a coordinated care plan, which means those patients are patients that have three or more comorbidities.” (P089) Older adults were another priority population. “It was our seniors, our vulnerable seniors’ population. Age 70 plus, those where the doc knew there were issue potentially loneliness, becoming shut-ins, patients with mobility access concerns.”(P010) Individuals with chronic diseases were also a priority for some practices. One ED reported, “we implemented a wellness check program to help the doctors out … we had contacted a lot of the physicians asking them if … they can provide us with lists of patients whether it was diabetes patients, hypertension patients, other chronic disease patients that we could call.”(P088).

Infants and prenatal parents were another priority group. Well-baby checks were important: “we generated a list of all the well-baby children, immunizations, and attached that to one individual to make sure that they were priority booking and no one was missed. P076.” Some programming for this population switched to virtual. “The dietitian … does a feeding solids program, so she’s now switching that to virtual … She’s hoping to then create the live version so that new moms can then log in wherever they are.” (P031).

Mental health and addictions were another priority area. “What we’ve realized is those are our vulnerable patients who are higher risk … whether it’s substance abuse or depression, or any other mental health related challenges. Our social work team really stepped up to connect with them to ensure that, from a from a health and well-being standpoint, that their care needs continue to be met. Even though we can’t actually bring them into the office.” (P036).

One ED encapsulated what was stated by many “I think senior, mental health, complex, medically complex, prenatal and I don’t know if I think of prenatal as vulnerable, but they were. … because once the babies are born, the babies have to come in for their shots.” (P037).

Some teams were focused on very specific populations depending on specific regional challenges. For example, several provided services to migrant agricultural workers. As one ED reported, “I’m going to say the migrant workers were vulnerable and they were scared of what was happening. We provided on-site … supports, emotional support. A lot of our NPs would stay and speak with the migrant workers. Some that were concerned and scared... At the time advocated with public health to follow up with them. We stayed on site and we listened to their fears and concerns.”(P084).

Other teams worked with the homeless population. “In the beginning we focused on homelessness. There’s nowhere in this community for homeless to go, and we were really focusing on what if somebody that’s homeless tests positive? Because they’re usually couch surfers so we didn’t want them just randomly staying at different people’s houses throughout the course of their COVID. Everybody really pulled together and got a little hotel B&B type situation for if that was to ever happen.”(P066).

The social determinants of health were important for many teams. “There’s the social determinants of health, if we highlight, for example, food. You know there were a lot of people who, for example, lost their jobs because their place of employment had to close with all the changes. Those are people who we could reach out to, and direct them to the services that are available, so that they can get food. Not only for nourishment, but it’s a peace of mind, it does give them better sense of peace. Then for those who are already anxious or are depressed that’s not an additional thing to worry about.”(P002) Similarly, one ED said, “food security was a big part of our immediate need when it hit because … all those doors where people would normally go over closed. We wanted to make sure that that was in place, basic sort of hierarchy of needs piece. “(P028).

Some teams made an effort to communicate with all their patients. “We kept in constant communication with our patient population. On a weekly or biweekly basis, letters were sent out or either mailed out or sent out electronically to inform our rostered patients of the changes that were happening within our organization, what the recommendations were with respect to masks to physical isolation, to ensuring the health and safety of family and friends. Then what their expectations should be, should they come into the office.” P036 Others reached out through social media and other channels, “we’ve been updating our website, and updating our social media with the updates around the office.”(P065).

### Stepping Up and Into New Roles

Some primary care teams became COVID-19 testing and assessment sites, requiring team members to take on new roles. “At the beginning of the pandemic, we actually started running the COVID-19 assessment centre. Originally our team had split off, half the team was working from our local clinic so that they were able to provide programs and services for urgent needs, and then the nurse practitioners and admin and an assistant were at the Family Health Team building doing swabs.”(P060).

Others deployed mobile assessment teams. For example, “our Family Health Team, stepped up and we provided a mobile assessment team for COVID-19 for the seasonal agricultural workers … We were contacted by public health on a Friday. We were involved in the emergency pandemic planning and within three hours we had nurse practitioners and physicians in the field. We did over 673 individual assessment encounters on the farm. In one month.”(P084).

EDs reported that team members quickly adapted to these redeployments. One commented, “initially for this area, everyone was doing their own testing for COVID-19, before the assessment center was open. I was really impressed with our team, how quickly they mobilized and set up to outdoor testing, and really embraced all the change.”(P073) Another stated, “when the pandemic hit and it was determined that we would be the best spot for the COVID-19 assessment centre, our team literally overnight transformed into the assessment centre, so that showed my team’s ability to be flexible.”(P060).

EDs were proud of their team members. One stated, “in the in the beginning when things were very uncertain, I had two of my nurses doing the COVID swabbing. One of my nurse’s husbands is palliative and my other nurse just had her first grandbaby and they both actually stayed away from their families for months while they were doing the testing … I’m proud of my team, they really stepped up.”(P066).

Other EDs stated that team members volunteered to help with COVID-19 assessment and patient management, or that their staff supported the local assessment centre. As one said, “my docs, I’ve got a number of them to take shifts the COVID assessment centre … One of my clinics that they provide low-risk obstetrics, they’ve actually been volunteering to attend maternity at the hospital to deal with COVID-positive mothers and support the birthing process through there. They’re doing some great stuff.”(P010).

## Discussion

The study provides important insights into primary care leader’s experiences during the rapid shift to virtual care during the early months of the COVID-19 pandemic restrictions. This change to virtual care came with challenges, including technological issues such as poor internet connectivity and cell phone service, some patients having difficulty accessing the required technology, and a lack of comfort with technology. Finding the right virtual care solution was another challenge, as was the cost of virtual care platforms. The significant learning curve that health care providers endured when shifting to virtual care may be in part because most primary care providers have had little to no education or training on virtual care. For some participants in our study, virtual care was less efficient and more time consuming that in-person care. Others reported security and privacy concerns with virtual care, as well as concerns about the quality of care provided virtually. EDs also indicated that virtual care was not always appropriate. It is unclear the long-term implications that virtual care has on quality of care. Further research will be needed to determine what types of care can optimally be delivered virtually and which require in-person care.

One of the major changes to primary care that resulted from the pandemic was the rapid implementation of virtual care or telehealth. EDs from Ontario’s AFHTO members reported a rapid shift to delivering care virtually. This shift has also been reported worldwide [8, 20] and for primary care practices in the United States [1, 3, 21–25], New Zealand [26], and the United Kingdom [27]. Virtual care was mostly provided through video or telephone [1, 3, 21–23, 26–28], with telephone being the preferred choice in certain circumstances [3, 29]. Interprofessional health care providers working at Ontario Family Health Teams also reported this change to virtual care [7].

The challenges of virtual care reported by EDs have also been found in other primary care practices. Poor internet access and cell phone reception are one of the commonly reported challenges [21, 26, 29]. The technological infrastructure needed to support virtual care is missing or underdeveloped in many locations [30, 31], an issue reported by many rural and remote EDs. A lack of confidence in using technology or familiarity with virtual care tools is another common barrier [22, 26, 27]. Additional challenges with virtual care included funding and cost issues [26] as well as privacy issues [26, 29, 32]. Virtual care was not appropriate for all conditions or all populations [26, 32].

EDs reported many benefits to virtual care, with some patients preferring this type of care. Similar to our findings, studies from New Zealand, the United Kingdom, and the United States reported that patients found that virtual care saved them time, did not require them to travel, reduced their stress, and minimized employment disruption [25, 26, 33]. Having safe, efficient, and timely care was also a benefit to virtual care, which was also found in primary care practices in the United Kingdom [27] and New Zealand [26].

With the transition to virtual care, EDs reported fewer “no shows” for appointments. This has been reported as one of the benefits of virtual visits [34, 35] and has been seen in other medical practices during the COVID-19 pandemic [36]. With virtual visits, patients do not have to travel to the primary care practice, do not need to pay for parking, and do not have to spend time in a waiting room [37]. The convenience of virtual care for patients was reported by many EDs.

Despite the shift to virtual care, EDs reported that in-person care continued for certain populations, confirming our findings with interprofessional health care providers [7]. This was also reported by practices in the United States [21, 23]. Teams continued to see vulnerable or complex patients in-person when required [24]. Early infant immunizations were continued [29]. Many teams also continued with home visits, particularly for homebound individuals, which other teams also report conducting [20, 38].

EDs reported that that some providers experienced a learning curve in transitioning to virtual care. The need for training health care providers on virtual care has been reported in the literature [25, 29, 31]. Other EDs expressed concerns over the quality of care being provided virtually. Currently there is a lack of consensus on the quality of care provided virtually [25]. Up until recently, health care providers did not receive training and education on virtual care [32, 39]. If virtual care continues to be offered, it will be important to ensure that health care providers are trained in its use, advantages, and disadvantages, so that they can provide optimal care to their patients [32, 39].

Continuing to provide support to at risk populations throughout the pandemic was important for Ontario’s interprofessional primary care teams. Wellness checks were one way teams supported at risk patients [40], which teams in the United States also report conducting [1, 21, 28, 31, 38]. Some teams became COVID assessment and testing centres, and primary care practices in other jurisdictions also fulfilled this role [20, 25].

The impact of virtual care is complex and experienced differently by different people. Further research is needed to determine when and for whom virtual care is appropriate and when in-person care is required. There is no one solution that works for everyone. We found that how virtual care is experienced is dependent upon the patient-provider relationship, something that has also been found in other studies [26, 27, 30]. It will also be important to ensure that virtual care does not disadvantage marginalized and traditionally underserved populations from accessing care. For example, virtual care may not be accessible to those with low incomes, who live in rural areas, are homeless, who are not technologically literature, who have a hearing impairment, or who do not have English or French as their first language [27, 30, 32, 33, 38].

While EDs discussed synchronous means of providing care, it should be noted that there are also asynchronous means available, and their use should be investigated moving forward. Further research should also examine what conditions and primary care services are best suited for different modes of delivery, including virtual care, in-person care, and home care. The preferences of different patient populations regarding the modes of care they prefer should also continue to be explored.

The findings presented here may help inform the design of future interventions in primary care. With virtual care, EDs indicated that virtual care requires support for infrastructure and technology, both for primary care teams and for the patients they serve. For patient populations at risk, EDs stated that adequate PPE for providers is essential so that in-person and home care can continue when required. As EDs mentioned some providers had difficulty with the transition to virtual care, training in virtual care and other modes of care delivery should be incorporated into health care professionals’ training.

### Strengths and limitations

This study was conducted in Ontario, Canada, the Canadian province with the highest population [41]. Interprofessional primary care teams belonging to AFHTO provide primary care services to more than 3.5 million individuals in over 200 communities throughout the province [42] and have operated for over a decade [15]. This provides an important context for examining how teams responded to the COVID-19 pandemic.

It is important to acknowledge that these interprofessional primary care teams are only one model of primary care and may not be representative of all primary care practices. While all EDs of AFHTO member practices were invited to take part in the study, not all consented to be interviewed. EDs who agreed to be interviewed may have had different experiences during the COVID-19 pandemic than those who were not interviewed. Additionally, health care provider and patient experiences and attitudes were indirectly reported by the EDs and may not be entirely accurate. While we received information on the geographical location of the primary care teams, we did not capture any demographic information on participating ED’s. Demographic data such as age, gender, length of time in their ED role could have offered insights into participant characteristics and ensured varying perspectives were included*.* It is also important to note that we did not return the transcripts to the EDs for comment or correction.

Nevertheless, EDs fill the most senior leadership position in these teams and are responsible for the day-to-day management of their primary care teams. They are therefore the individuals with the greatest knowledge of how the COVID-19 pandemic affected the operations of their primary care teams. A strength of this study was the in-depth interviews conducted with 71 of these senior leaders.

## Conclusions

This study provides a snapshot of how AFHTO member interprofessional primary health care teams in Ontario, Canada, responded to the COVID-19 pandemic. Teams rapidly mobilized to delivering the majority of their care virtually, primarily through telephone and video encounters, while continuing to provide in-person and home care as required. Additionally, teams found innovative ways to deliver care and many converted group programs to virtual delivery. Major challenges to virtual care included technological infrastructure, including lack of internet and cell phone access as well as unfamiliarity or uncertainty with virtual platforms. Advantages to virtual care included convenience and time savings. Virtual care will likely continue to be an important mode of primary care delivery moving forward, and its advantages and disadvantages will need to be considered. Efforts will need to be made to ensure that an increase in virtual care does not lead to an increase health and health care disparities.

## Data Availability

The qualitative dataset is available from the corresponding author on reasonable request.

## Abbreviations

AFHTO: Association of Family Health Teams of Ontario
FHT: Family Health Team
ED: Executive Director
PPE: Personal Protective Equipment
